# Exploring the landscape of Parkinson’s disease transcriptomics: a quantitative review of research progress and future directions

**DOI:** 10.3389/fnagi.2025.1505374

**Published:** 2025-05-21

**Authors:** Yiran Wang, Tong Li, Xiaoqing Zhang, Haoran Tai, Weihong Li, Bingyin Su

**Affiliations:** ^1^Chengdu University of Traditional Chinese Medicine, Chengdu, China; ^2^Development and Regeneration Key Lab of Sichuan Province, Department of Histology and Embryology, Chengdu Medical College, Chengdu, China; ^3^Department of Pathology, Chengdu Medical College, Chengdu, China; ^4^Hospital of Chengdu University of Traditional Chinese Medicine, Chengdu, China; ^5^Sichuan College of Traditional Chinese Medicine, Mianyang, China

**Keywords:** Parkinson’s disease, transcriptomics, bibliometric analysis, biomarkers, single-cell transcriptomics, spatial transcriptomics, neurodegeneration

## Abstract

**Introduction:**

This study leverages bibliometric analysis to uncover the research landscape and spotlight emerging trends in the field of Parkinson’s disease (PD) transcriptomics.

**Methods:**

The relevant literature on Parkinson’s disease and transcriptomics was retrieved from the Web of Science Core Collection database. Bibliometric analysis was conducted using VOSviewer, CiteSpace, and the Bibliometrix R package.

**Results:**

A total of 208 research articles were retrieved from January 2011 to March 2025. The number of publications has shown a steady increase, particularly from 2020 to 2024, with an average annual publication rate of 29 articles during this period. The United States and China were the leading countries in terms of publication counts, while the University of Luxembourg and McGill University were the top contributing institutions. The most impactful journals included “Nature Communications” and “NPJ Parkinson’s Disease.” The co-occurrence analysis of keywords revealed that “Parkinson’s disease,” “transcriptomics,” “neurodegeneration,” and “biomarkers” are current research hotspots. Citation burst analysis identified key references related to genetics, transcriptomics, and data analysis tools that have significantly influenced the field.

**Conclusion:**

This study offers the first comprehensive bibliometric analysis of Parkinson’s disease (PD) transcriptomics research from 2011 to 2025. We reveal a significant surge in research activity, particularly since 2020, driven by advancements in single-cell and spatial transcriptomics. The United States and China lead in publication output, with key contributions from the University of Luxembourg and McGill University. Research hotspots include neuroinflammation, biomarker discovery, and machine learning applications, indicating a shift toward translational research. However, challenges such as data heterogeneity and high biomarker validation failure rates persist. Future research should focus on standardizing methodologies and enhancing clinical relevance. Strategic directions include multi-omics integration, global collaboration, and linking transcriptomic signatures to clinical outcomes, aiming to improve early diagnosis and personalized therapies for PD.

## 1 Introduction

Parkinson’s disease (PD) is a prevalent neurodegenerative disorder characterized by the progressive loss of dopaminergic neurons in the substantia nigra of the midbrain and the formation of Lewy bodies (Titova et al., 2017; Dorsey et al., 2018), with primary clinical manifestations including bradykinesia, tremors, rigidity, and balance impairments (Bloem et al., 2021; Armstrong and Okun, 2020; Keo et al., 2021; Fereshtehnejad et al., 2019). Although the exact etiology of PD remains unclear (Bellou et al., 2016; Stoker and Greenland, 2018), research suggests that its pathogenesis involves genetics, environmental factors, and complex molecular and cellular processes (Cacabelos, 2017; Miller and O’Callaghan, 2015; Saracchi et al., 2014).

In recent years, transcriptomics, as a vital tool for studying the molecular mechanisms of PD (Kia et al., 2021), has been widely applied. Transcriptomics (Tiklova et al., 2020), through high-throughput sequencing technologies, quantitatively analyzes RNA expression across the entire genome, revealing disease-related changes in gene expression, gene regulatory networks, and potential biomarkers. This is of significant importance for understanding the pathophysiological mechanisms of PD (Ye et al., 2023) and for developing new therapeutic strategies (Van Oekelen et al., 2021).

Despite the many important findings in PD research through transcriptomics, the research hotspots, trends, and key contributors in this field have not yet been systematically summarized. Bibliometric analysis (Ma et al., 2021), as a quantitative analysis method, can reveal the dynamics of research fields, hotspots, and the contributions of major research institutions and countries by statistically analyzing a vast amount of literature data. This is of significant reference value for the strategic planning and resource allocation of scientific research.

The aim of this study is to systematically analyze research literature related to Parkinson’s disease and transcriptomics, revealing research trends, hotspots, and key contributors in the field. We will retrieve relevant literature from the Web of Science Core Collection database and conduct a quantitative analysis using bibliometric analysis methods, including annual publication volume, contributions by countries and institutions, journal distribution, keyword co-occurrence analysis, and research hotspot analysis. This study will provide valuable information and references for transcriptomics research in Parkinson’s disease, which may offer direction and inspiration for future research.

## 2 Methods

### 2.1 Search strategy

**Figure 1:**
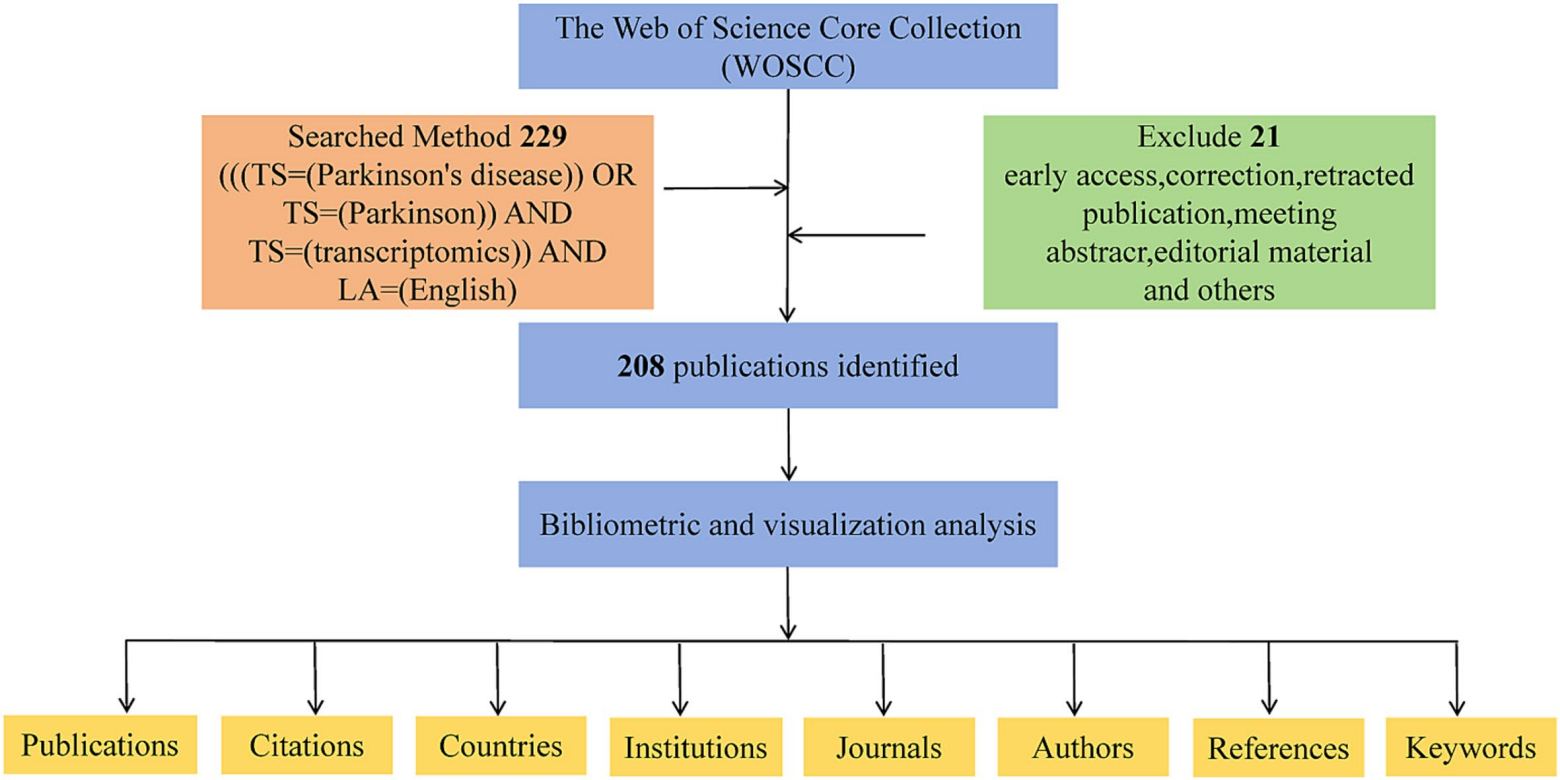
A detailed flowchart illustrating the systematic steps of the search strategy used in screening publications for research.

The study conducted a comprehensive search in the Web of Science Core Collection (WoSCC), a multidisciplinary and comprehensive database with an extensive citation network. The search was conducted without temporal limitations, focusing solely on documents categorized as “Articles” or “Reviews.” On March 20, 2025, two researchers independently conducted literature searches. The search strategy employed was: ((TS = (Parkinson’s disease) OR TS = (Parkinson)) AND TS = (transcriptomics)) AND LA = (English), with the entire retrieval process graphically represented in Figure 1.

### 2.2 Literature analysis

Data analysis and management were performed using VOSviewer 1.6.20, CiteSpace 6.3.R1, Bibliometrix 4.4.1, and Microsoft Office Excel 2010. VOSviewer 1.6.20 (van Eck and Waltman, 2010) was utilized for visualizing highly co-cited literature and author co-occurrence, as well as for analyzing national and institutional publication, journal analysis, author analysis, keyword co-occurrence, and co-cited reference analysis. CiteSpace 6.3.R1 (Synnestvedt et al., 2005) generated visual maps providing a detailed summary analysis of journal reference emergence maps. Bibliometrix 4.4.1 (Aria and Cuccurullo, 2017) was employed to perform a thematic analysis and to map out a global distribution network of scholarly publications pertaining to transcriptomics in the context of Parkinson’s disease.

## 3 Results

### 3.1 Analysis of annual publications and publication trends

**Figure 2:**
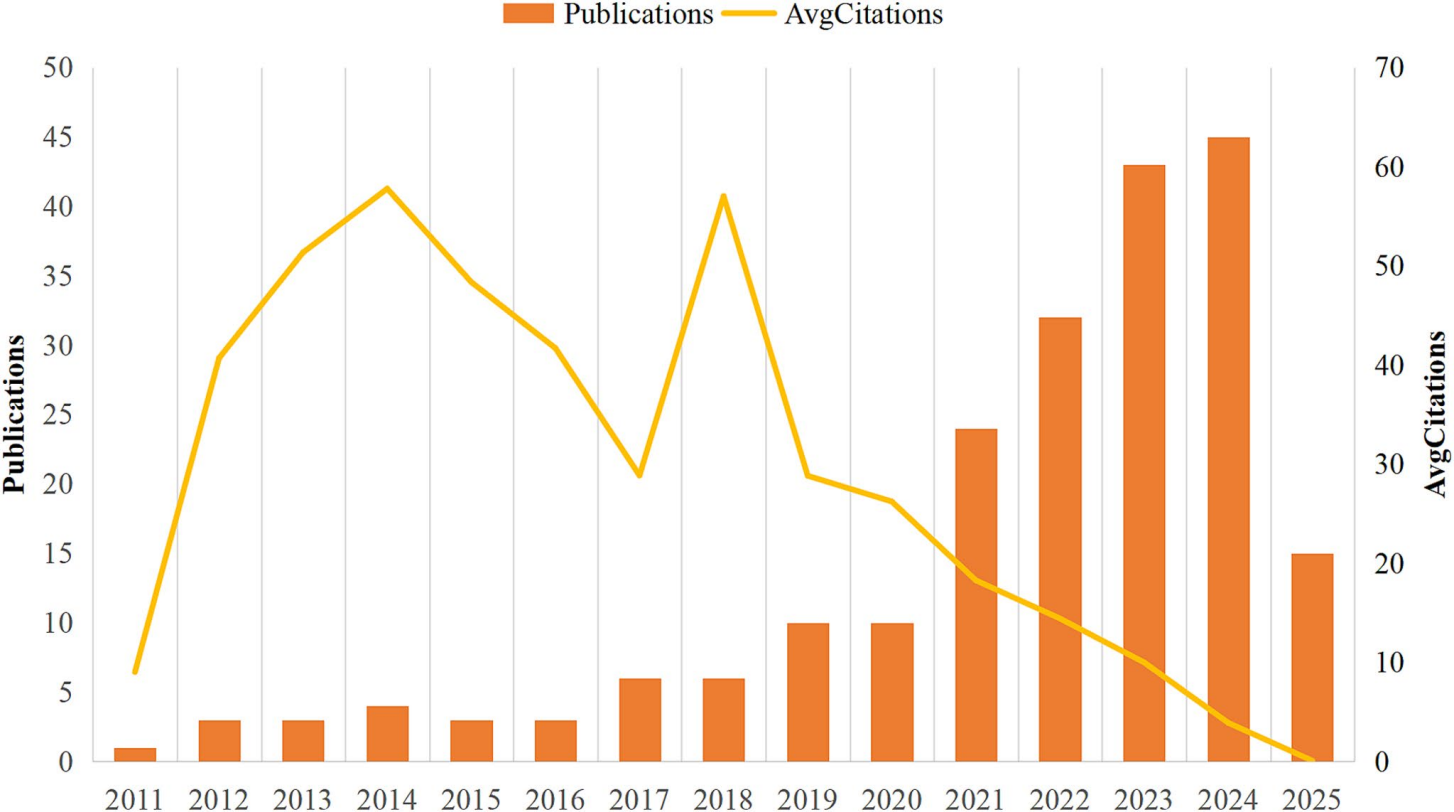
A graphical representation of the annual research output in the field of Parkinson’s disease transcriptomics.

According to our search criteria, a total of 208 research articles on the intersection of Parkinson’s disease and transcriptomics were retrieved from January 2011 to March 2025. Figure 2 presents the annual publication trend and average citations per publication. The publication trend can be segmented into distinct phases.

From 2011 to 2016, the field was in its early stages, with an average annual publication rate of approximately 2.8 articles. This period was characterized by sporadic research activities and limited scholarly attention. The average citations per publication showed a general upward trend during this phase, peaking at 57.75 in 2014, indicating the growing influence and recognition of research in this area.

In 2017, there was a noticeable increase in publications, with 6 articles published, marking the beginning of a period of steady growth. The average citations per publication also continued to rise, reaching 57.00 in 2018. However, from 2018 to 2019, although the publication output continued to increase, reaching 10 articles in 2019, the average citations per publication experienced a decline, possibly reflecting the rapid expansion of research without a corresponding immediate impact.

The period from 2020 to 2023 witnessed a significant surge in publication numbers, with an average annual increase to about 29 articles, peaking at 43 articles in 2023 and 45 articles in 2024. This substantial growth suggests that research at the nexus of Parkinson’s disease and transcriptomics is gaining prominence within the broader field of neuroscience. The average citations per publication showed a fluctuating trend during this period, with a notable decrease in 2025, which might be attributed to the recency of publications and the time lag in citation accumulation.

### 3.2 Country and institutional analysis

**Figure 3:**
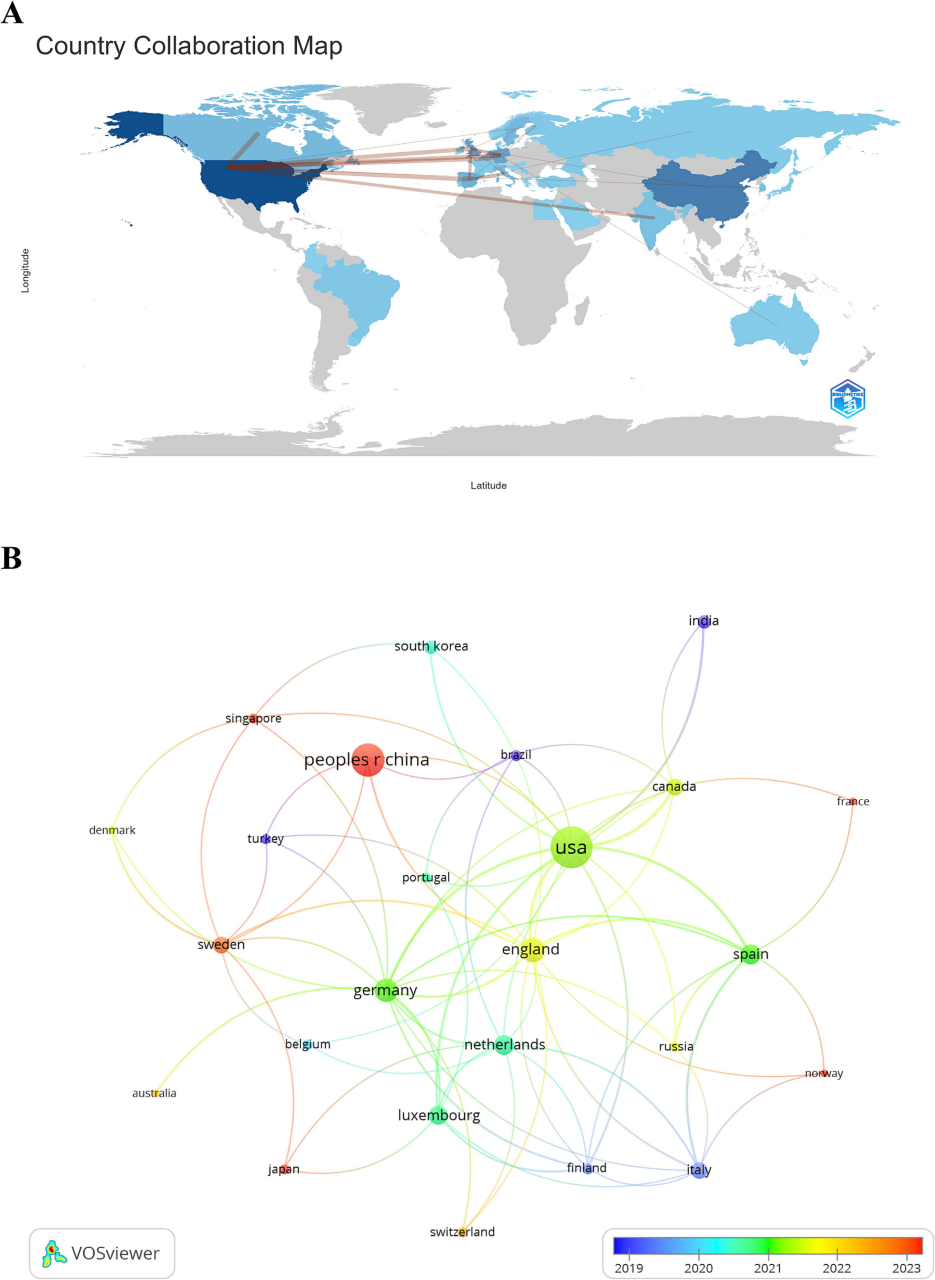
A visual depiction of the geographical distribution **(A)** and a map-based visualization of countries **(B)** contributing to research on transcriptomics in Parkinson’s disease.

Publications originated from 39 countries and 412 institutions. The top ten contributing countries are distributed across Europe, North America, and Asia, with a significant concentration in Europe (=8) and North America (=2) (Table 1). The United States leads with the highest number of publications (=53), demonstrating its dominant position in the field. This is followed by China (=33), the United Kingdom (=19), Germany (=17), and Spain (=12). The combined publications from these five countries account for over half of the total (54.25%). Subsequently, we filtered and visualized 25 countries based on a publication count of two or more, constructing a collaborative network based on the number and relationships of publications per country (Figures 3A,B). Notably, the United States has connections with 15 countries, while China has links with five countries. More extensive cooperation with additional countries should be pursued in the next steps to accelerate the development of this research field.

**Table 1:** Top 10 countries/institutions on research of Parkinson’s disease in transcriptomics.

Rank	Country	Counts	Institution	Counts
1	USA	53 (25.48%)	University of Luxembourg	11 (5.29%)
2	China	33 (15.87%)	McGill University	6 (2.88%)
3	England	19 (9.13%)	University College London	6 (2.88%)
4	Germany	17 (8.17%)	Karolinska Institute	5 (2.40%)
5	Spain	12 (5.77%)	Maastricht University	5 (2.40%)
6	Netherlands	12 (5.77%)	Capital Medical University	4 (1.92%)
7	Luxembourg	11 (5.29%)	Lund University	4 (1.92%)
8	Sweden	9 (4.33%)	University of Barcelona	4 (1.92%)
9	Italy	9 (4.33%)	University of Cambridge	4 (1.92%)
10	Canada	9 (4.33%)	University of Oxford	4 (1.92%)

**Figure 4:**
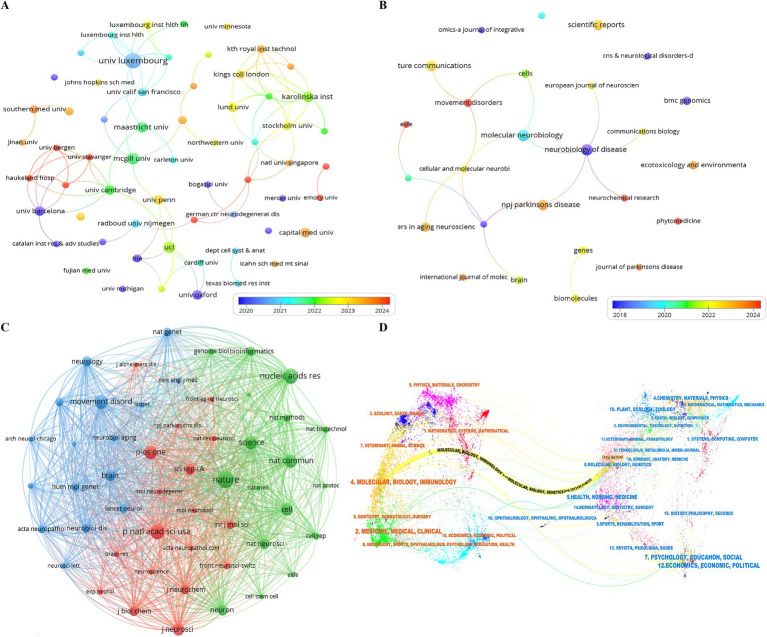
A comprehensive visualization of research on transcriptomics in Parkinson’s disease, encompassing **(A)** Institutions, **(B)** Journals, **(C)** Co-Cited Journals, and **(D)** A dual-map overlay highlighting the interconnections between journals.

The top ten institutions are in seven countries (Luxembourg, Canada, the United Kingdom, Sweden, the Netherlands, China, and Spain). Institutions with ≥5 relevant publications are University of Luxembourg (=11, 5.29%), McGill University (=6, 2.88%), University College London (=6, 2.88%), Karolinska Institute (=5, 2.40%), and Maastricht University (=5, 2.40%). We then selected 61 institutions for visualization based on a minimum publication count of two and constructed a collaborative network based on the number and relationships of publications per institution (Figure 4A). As shown in Figure 4A, University of Luxembourg has very close collaborations with the Harvard Medical School, Leiden University, Luxembourg Institute of Health, and University of California, San Diego. Additionally, we observed that Capital Medical University has the highest publication count in China, while Southern Medical University has the highest citation count.

In summary, the collaborative networks among countries and institutions reveal global trends in cooperation and leadership, providing multifaceted perspectives and methodologies for addressing complex scientific issues. This is crucial for advancing scientific development, improving patients’ quality of life, and fostering innovation in research.

### 3.3 Journals and co-cited journals

Publications concerning transcriptomics in Parkinson’s disease have appeared across 106 journals. Molecular Neurobiology and Neurobiology of Disease both led with the highest number of publications (=6, 2.88%), followed by Molecular Neurobiology and Neurobiology of Disease both led with the highest number of publications (=6, 2.88%), followed by Nature Communications, NPJ Parkinson’s Disease, and Scientific Reports (=5, 2.40%). Among the top 15 journals, Nature Communications had the highest impact factor (IF = 14.7007), closely followed by Brain (IF = 12.8458). Subsequently, we filtered 26 journals based on a minimum of two relevant publications and plotted a journal network diagram (Figure 4B). Figure 4B illustrates active citation relationships among Neurobiology of Disease, Molecular Neurobiology, and NPJ Parkinson’s Disease, indicating the close connections of Parkinson’s disease transcriptomics research with other fields such as neuroscience, molecular biology, genetics, bioinformatics, and clinical medicine.

As shown in Table 2, among the top 15 most-cited journals, six journals have been cited over 230 times. Nature was the most cited (total citations = 345), followed by Nucleic Acids Research and Proceedings of the National Academy of Sciences of the United States of America (total citations = 270 each), Movement Disorders (total citations = 248), Science (total citations = 234), and PLOS ONE (total citations = 231). These high citation counts reflect the central role and broad influence of these journals in academia. They also reveal the interrelationships among research literature and the flow of knowledge, which is crucial for understanding the depth and breadth of the research field. Additionally, a co-citation network was plotted after filtering journals with a minimum co-citation count of 50 (Figure 4C). As shown in Figure 4C, these journals have positive co-citation relationships with other journals, indicating their significant influence and close academic connections in related research areas.

**Table 2:** Top 15 journals/co-cited journals for Parkinson’s disease in transcriptomics.

Rank	Journal	Counts	IF	Q	Co-cited Journal	Co-citation	IF	Q
1	Molecular Neurobiology	6	4.436	1	Nature	345	50.5012	1
2	Neurobiology of Disease	6	5.7181	1	Nucleic Acids Research	270	15.2088	1
3	Nature Communications	5	14.7007	1	Proceedings of the National Academy of Sciences of the United States of America	270	9.4006	1
4	NPJ Parkinson’s Disease	5	6.7	1	Movement Disorders	248	7.4001	1
5	Scientific Reports	5	3.8001	1	Science	234	49.6291	
6	Frontiers in Aging Neuroscience	4	4.7467	2	PLOS ONE	231	2.8997	1
7	Biomolecules	3	4.8534	1	Nature Communications	222	14.7007	1
8	BMC Genomics	3	3.9159	2	Cell	209	45.4993	1
9	Brain	3	12.8458	1	The Journal of Neuroscience	199	6.709	1
10	Cells	3	5.4147	2	Brain	185	12.8458	1
11	Ecotoxicology and Environmental Safety	3	6.3996	1	Scientific Reports	173	3.8001	1
12	Genes	3	2.9643	2	Neuron	171	14.7007	1
13	Movement Disorders	3	8.5573	1	Nature Neuroscience	164	21.0932	1
14	BMC Medical Genomics	2	2.0431	3	Neurology	160	7.7002	1
15	Cellular and Molecular Life Sciences	2	6.5287	1	Journal of Biological Chemistry	156	4.8097	2

The double map overlay of journals shows the citation relationships between citing and cited journals. On the left are clusters of citing journals, and on the right are clusters of cited journals. As shown in Figure 4D, the yellow paths represent the primary citation routes, indicating that publications in Molecular/Biology/Immunology journals are predominantly cited by literature in Molecular/Biology/Genetics journals. The analysis of journals and their co-cited journals not only reveals the patterns of academic exchange and knowledge flow in Parkinson’s disease transcriptomics research but also plays a significant role in promoting in-depth research, enhancing the quality and impact of research, and fostering interdisciplinary collaboration.

### 3.4 Authors and co-cited authors

**Figure 5:**
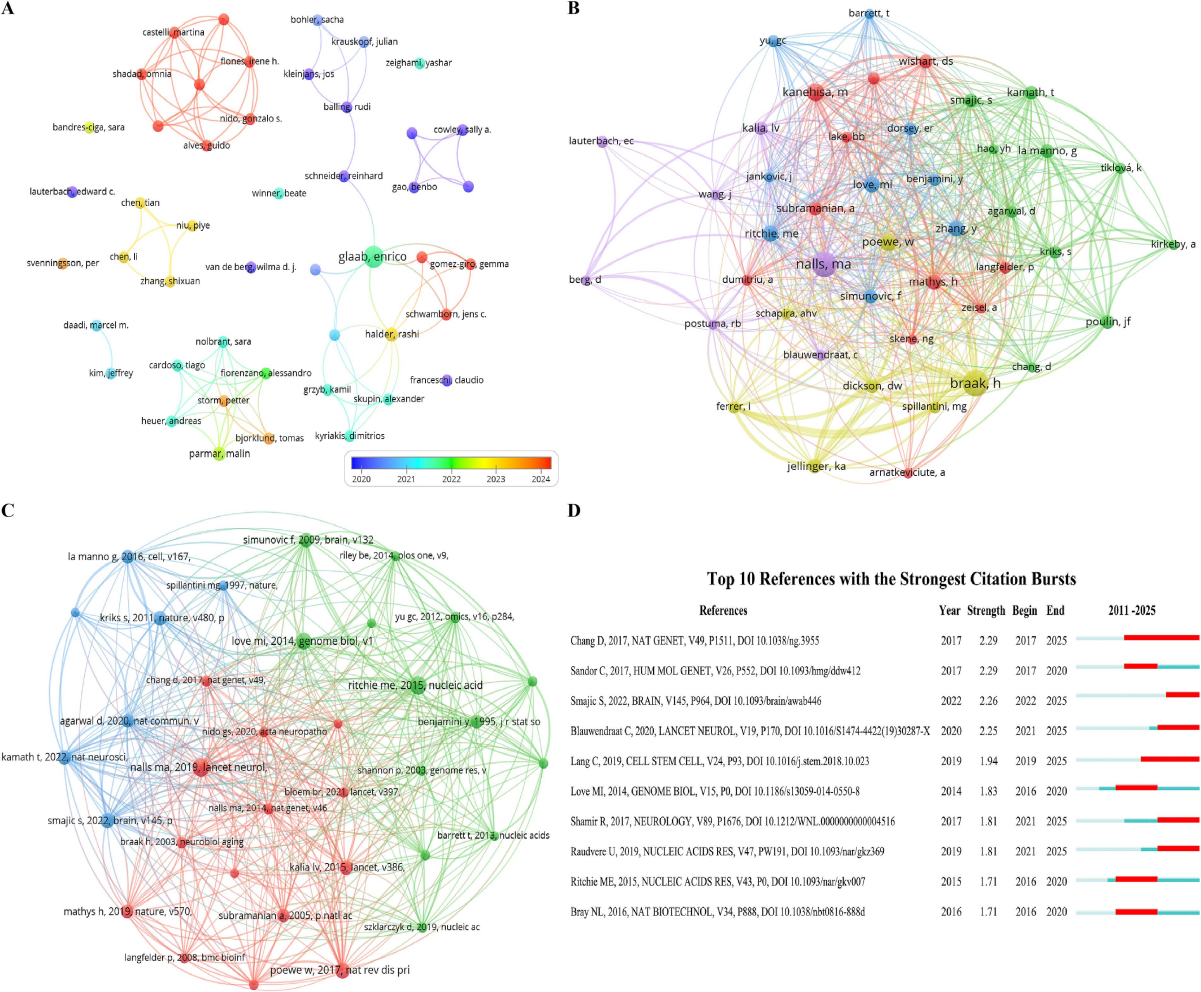
A focused visualization of key elements in Parkinson’s disease transcriptomics research, featuring **(A)** Authors, **(B)** The co-cited authors network, **(C)** Co-cited references, and **(D)** The top 12 references exhibiting strong citation bursts, marked by a red bar indicating years of notably high citation activity.

A total of 1,181 authors have contributed to Parkinson’s disease transcriptomics research. In the top 10, Glaab, Enrico from the University of Luxembourg ranks first with 7 papers. Halder, Rashi from the University of Bonn and Parmar, Malin from Lund University each have 3 papers, while the remaining seven authors have 2 each (Table 3). We constructed a collaboration network (Figure 5A) based on authors with two or more papers. Multiple authors have close collaborations. Glaab, Enrico works closely with Halder, Rashi, Parmar, Malin, Alves, Guido, Balling, Rudi, and others, and actively collaborates with Bandres-Ciga, Sara, Bjorklund, Tomas, Bohler, Sacha, and others.

**Table 3:** Top 10 authors and co-cited authors for Parkinson’s disease in transcriptomics.

Rank	Author	Counts	Co-cited Author	Citations
1	Glaab, Enrico	7	Braak, H	38
2	Halder, Rashi	3	Nalls, Ma	35
3	Parmar, Malin	3	Kanehisa, M	21
4	Alves, Guido	2	Poewe, W	20
5	Balling, Rudi	2	Love, Mi	19
6	Bandres-Ciga, Sara	2	Ritchie, Me	18
7	Bjorklund, Tomas	2	Zhang, Y	18
8	Bohler, Sacha	2	Mathys, H	17
9	Buttini, Manuel	2	Jellinger, Ka	16
10	Cardoso, Tiago	2	Kalia, Lv	15

Among the 10,143 co-cited authors, four have over 20 co-citations (Table 3). The most co-cited is Braak, H with 38, followed by Nalls, Ma (Priem et al., 2010), Kanehisa, M (Kim et al., 2014), and Poewe, W (Hussein et al., 2023). After filtering for a minimum co-citation count of 10, a co-citation network graph was plotted (Figure 5B). As shown in Figure 5B, co-citation analysis reveals the intersections between different research fields, and such collaborations may foster interdisciplinary research and the development of new theories. The analysis of author and co-citation networks helps researchers quickly identify key figures and research teams in the field, facilitating the tracking of cutting-edge issues.

### 3.5 Co-cited references

There are 12,183 co-cited references in the research on Parkinson’s disease transcriptomics, indicating a solid academic foundation and a broad range of knowledge sources for this field of study. Among the top 10 co-cited articles (Table 4), each has been cited at least 12 times, with one article receiving over 18 citations. A co-citation network graph (Figure 5C), constructed by selecting references with a co-citation count greater than or equal to 8, reveals the interconnectivity among these documents.

**Table 4:** Top 10 co-cited references on research of Parkinson’s disease in transcriptomics.

Rank	Co-cited references	Citations
1	Identification of novel risk loci, causal insights, and heritable risk for Parkinson’s disease: a meta-analysis of genome-wide association studies (Nalls et al., 2019)	18
2	Limma powers differential expression analyses for RNA-sequencing and microarray studies (Ritchie et al., 2015)	18
3	Moderated estimation of fold change and dispersion for RNA-seq data with DESeq2 (Love et al., 2014)	16
4	Parkinson disease (Poewe et al., 2017)	15
5	Single-cell sequencing of human midbrain reveals glial activation and a Parkinson-specific neuronal state (Smajić et al., 2022)	14
6	Parkinson’s disease (Kalia and Lang, 2015)	13
7	Single-cell genomic profiling of human dopamine neurons identifies a population that selectively degenerates in Parkinson’s disease (Kamath et al., 2022)	13
8	Dopamine neurons derived from human ES cells efficiently engraft in animal models of Parkinson’s disease (Kriks et al., 2011)	13
9	Gene expression profiling of substantia nigra dopamine neurons: further insights into Parkinson’s disease pathology (Simunovic et al., 2009)	13
10	A single-cell atlas of the human substantia nigra reveals cell-specific pathways associated with neurological disorders (Agarwal et al., 2020)	12

According to Figure 5C, “Identification of novel risk loci, causal insights, and heritable risk for Parkinson’s disease: a meta-analysis of genome-wide association studies” and “Limma powers differential expression analyses for RNA-sequencing and microarray studies” have active co-citation relationships. Nalls’ work offers a multi-omics analysis framework for Parkinson’s disease transcriptomics, linking genetics and transcriptomics to highlight the importance of data integration. Ritchie’s study provides the Bioconductor package, a powerful data analysis tool.

Additionally, “Limma powers differential expression analyses for RNA-sequencing and microarray studies” and “Moderated estimation of fold change and dispersion for RNA-seq data with DESeq2” also have active co-citation relationships. “Limma” and “DESeq2” provide statistical methods and tools for accurate gene expression analysis in Parkinson’s disease.

Other references contribute to Parkinson’s disease research and treatment in various ways, including comprehensive reviews, molecular mechanisms, neuroinflammation, neuroprotection, and stem cell therapy strategies. Transcriptomics studies are crucial for understanding disease mechanisms and finding potential therapeutic targets, driving the field towards deeper and broader development.

### 3.6 Citation burst analysis of references

Reference with citation bursts refers to those references that are frequently cited by scholars in a certain field over a period of time. In our study, 10 references with strong citation bursts were identified by CiteSpace (Figure 5D). As shown in Figure 5D, every bar represents a year, and the red bar represents strong citation burstiness. Citation bursts for references appeared as early as 2016 and as late as 2025. The reference with the strongest citation burst (strength = 2.29) was titled “A meta - analysis of genome - wide association studies identifies 17 new Parkinson’s disease risk loci,” published in Nature Genetics by Diana Chang et al., with citation bursts from 2017 to 2025. At the same time, the reference with the same burst intensity is titled “Transcriptomic profiling of purified patient - derived dopamine neurons identifies convergent perturbations and therapeutics for Parkinson’s disease,” authored by Cynthia Sandor et al., with citation bursts from 2017 to 2020. Overall, the burst strength of these 10 references ranged from 1.71 to 2.29, and their endurance strength ranged from 3 to 8 years. Table 5 summarizes the relevance between the main research contents of these 10 articles and the research field of Parkinson’s disease transcriptomics according to the order of the literature in Figure 5D.

**Table 5:** Top 10 references with strong citation bursts.

Rank	Title	Strength	Association with Parkinson’s disease in transcriptomics
1	A meta-analysis of genome-wide association studies identifies 17 new Parkinson’s disease risk loci (Chang et al., 2017)	2.29	Identify 17 new Parkinson’s risk loci, offering potential gene targets for transcriptomics.
2	Transcriptomic profiling of purified patient-derived dopamine neurons identifies convergent perturbations and therapeutics for Parkinson’s disease (Sandor et al., 2017)	2.29	Transcriptome analysis identified convergent perturbations and treatments for Parkinson’s disease.
3	Single-cell sequencing of human midbrain reveals glial activation and a Parkinson-specific neuronal state (Smajić et al., 2022)	2.26	Single cell sequencing provides evidence for cell heterogeneity and specific transcriptome changes in Parkinson’s disease.
4	The genetic architecture of Parkinson’s disease (Blauwendraat et al., 2020)	2.25	Provide genetic background info, identify key genes and pathways to guide transcriptomic research.
5	Single-Cell Sequencing of iPSC-Dopamine Neurons Reconstructs Disease Progression and Identifies HDAC4 as a Regulator of Parkinson Cell Phenotypes (Lang et al., 2019)	1.94	Reconstruct Parkinson’s progression, identifying key factor HDAC4.
6	Moderated estimation of fold change and dispersion for RNA-seq data with DESeq2 (Love et al., 2014)	1.83	Provide DESeq2 for differential expression analysis.
7	Analysis of blood-based gene expression in idiopathic Parkinson disease (Shamir et al., 2017)	1.81	Blood gene expression analysis clues aid in finding Parkinson’s - related peripheral biomarkers.
8	g:Profiler: a web server for functional enrichment analysis and conversions of gene lists (2019 update) (Raudvere et al., 2019)	1.81	Provide g:Profiler for functional enrichment analysis.
9	Limma powers differential expression analyses for RNA-sequencing and microarray studies (Ritchie et al., 2015)	1.71	Provide limma for differential expression analysis.
10	Erratum: Near-optimal probabilistic RNA-seq quantification (Bray et al., 2016)	1.71	Provide gene expression quantification basis, ensuring reliability and accuracy of subsequent analysis.

These references cover multiple research directions in Parkinson’s disease, including genetics, transcriptomics, cellular heterogeneity, disease progression, and data analysis, which are closely related to the molecular mechanisms, pathological processes, and potential therapeutic strategies of Parkinson’s disease. Additionally, they provide advanced data analysis tools, such as DESeq2, Limma, and g:Profiler, as well as single-cell RNA sequencing technology, offering crucial technical support for Parkinson’s disease transcriptomics research. These studies not only enhance the understanding of the complexity of Parkinson’s disease but also provide important scientific evidence for the development of new treatments and the improvement of clinical treatment efficiency.

### 3.7 Hotspots and Frontiers

Keyword co-occurrence analysis swiftly identifies research hotspots within a specific domain. Table 6 presents the top 20 most frequently occurring keywords in Parkinson’s disease transcriptomics research. Notably “Parkinson’s disease,” “Transcriptomic,” and “Neurodegeneration” have emerged more than 15 times highlighting the primary research focus within this field.

**Table 6:** Top 20 keywords on research of Parkinson’s disease in transcriptomics.

Rank	Keywords	Counts
1	Parkinson’s disease	58
2	Transcriptomics	36
3	Neurodegeneration	16
4	Proteomics	13
5	Alzheimer’s disease	12
6	Neurodegenerative diseases	7
7	Neurotoxicity	7
8	Rna-Seq	7
9	Alpha-Synuclein	6
10	Aging	5
11	Huntington’s disease	5
12	Parkinson disease	5
13	Rna Sequencing	5
14	Machine Learning	4
15	Metabolomics	4
16	Microglia	4
17	Network Analysis	4
18	Neuroinflammation	4
19	Substantia Nigra	4
20	Amyotrophic Lateral Sclerosis	3

**Figure 6:**
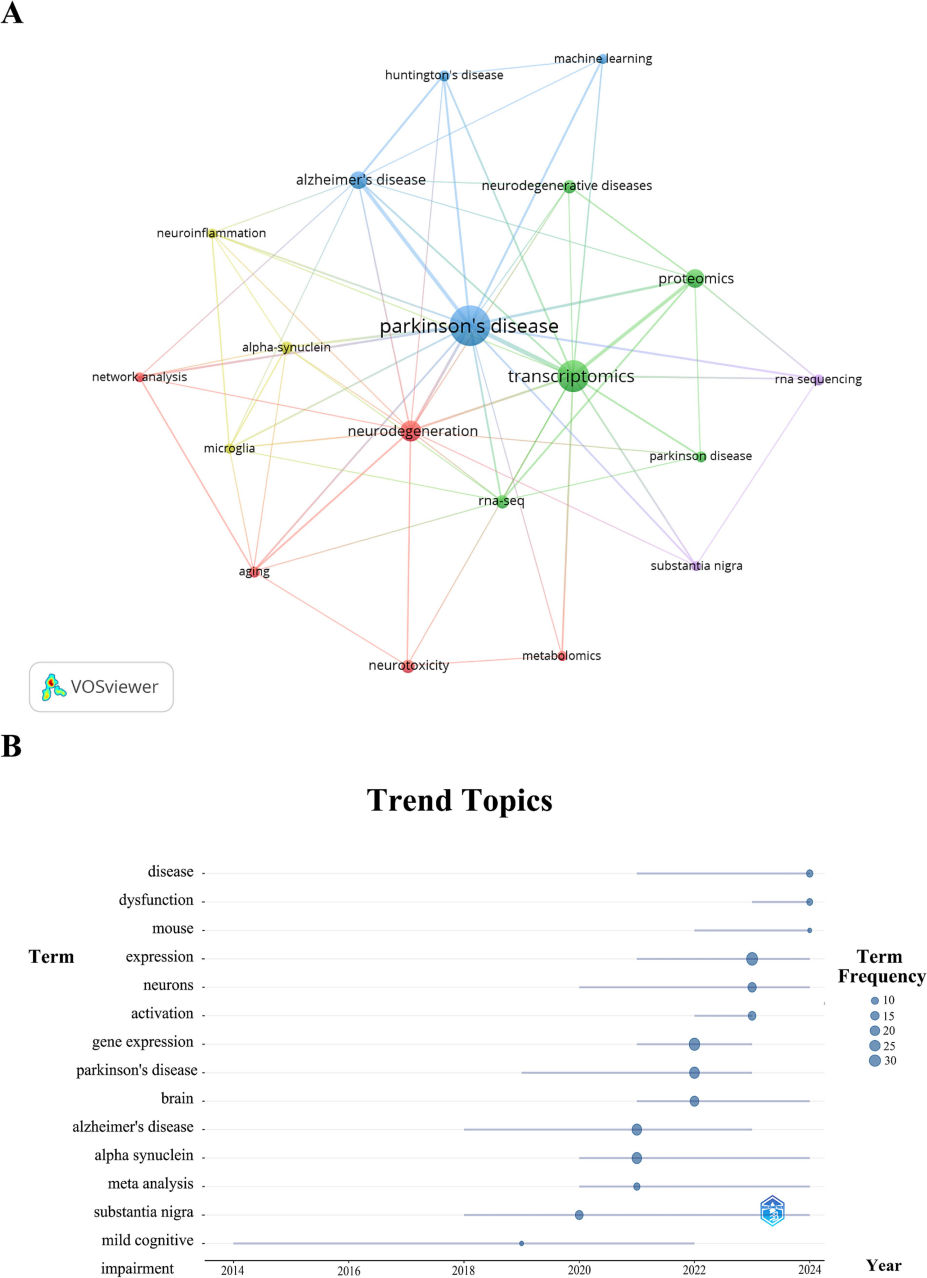
An analysis of keyword clustering **(A)** and trend topics **(B)** within the domain of Parkinson’s disease transcriptomics research.

By filtering for keywords with occurrences of four or more and applying cluster analysis via VOSviewer, we visualized the relationships, where thicker lines between nodes indicate stronger connections. The analysis revealed five distinct clusters, each representing a research direction. The red cluster encompasses keywords such as “Aging,” “Metabolomics,” “Network analysis,” “Neurodegeneration,” and “Neurotoxicity,” mainly involving studies on the pathological mechanisms and disease progression of Parkinson’s disease. The green cluster encompasses terms like “Neurodegenerative disease,” “Parkinson disease,” “Proteomics,” “Rna - seq,” and “Transcriptomics,” primarily involving molecular - level research on Parkinson’s disease, including gene expression, proteomics, and transcriptomics analyses. The blue cluster features “Alzheimer’s disease,” “Huntington’s disease,” “Machine learning,” and “Parkinson’s disease,” mainly involving comparative studies of neurodegenerative diseases and the application of machine learning in these diseases. The yellow cluster centers on “Alpha - synuclein,” “Microglia,” and “Neuroinflammation,” mainly involving studies on neuroinflammation in Parkinson’s disease and research on specific proteins. Finally, the purple cluster includes “Rna sequencing” and “Substantia nigra,” mainly involving RNA - sequencing studies on specific brain regions in Parkinson’s disease. (Figure 6A)

Trend topic analysis of these keywords (Figure 6B) indicates that from 2014 to 2020, research predominantly concentrated on “Parkinson’s disease,” “neurons,” “substantia nigra,” “expression,” and “activation.” Since 2020, there has been a marked shift towards exploring “Alzheimer’s disease,” “alpha synuclein,” “meta analysis,” and “mouse,” with a focus on keywords related to neurodegenerative diseases associated with Parkinson’s disease, specific proteins, analytical methods, and animal models.

Furthermore, the keywords “Parkinson’s disease,” “gene expression,” and “substantia nigra” have been consistently prominent over the last 5 years (2020–2024), suggesting that they are likely to represent current research hotspots in Parkinson’s disease transcriptomics. This analysis reveals an expansion of research focus from basic disease mechanisms to broader neurodegenerative diseases and multi - omics research areas, reflecting the interdisciplinary development trend of the field. This has significant implications for deepening our understanding of the pathological mechanisms of Parkinson’s disease and developing new treatments.

## 4 Discussion

### 4.1 General information discussion

This article quantitatively analyzes the research field of Parkinson’s disease and transcriptomics using bibliometric methods. From 2011 to March 2025, a total of 208 studies have been published, with an average annual publication rate of 29 articles between 2020 and 2023, showing a steady increase in the number of publications in 2023 and 2024. This indicates that the field has become one of the hot topics in neuroscience research and is expected to continue expanding and deepening in the coming years.

Publications have originated from 412 institutions across 39 countries, highlighting the global nature of this research area. The United States leads the national ranking with 53 publications, accounting for 25.5% of the total, followed closely by China with 33 publications (15.9%). Together, the United States and China contribute to approximately half of the total publications.

In terms of institutional rankings, the University of Luxembourg tops the list with 11 publications, followed by McGill University with 6. The collaboration network of these leading institutions demonstrates a trend of international cooperation, which is crucial for advancing scientific development, promoting knowledge sharing, and enhancing research efficiency and innovation capabilities.

Publications are spread across 106 journals, with “Molecular Neurobiology” and “Neurobiology of Disease” having the highest number of publications, while “Nature” is the most frequently cited journal. The journal network diagram reveals active citation relationships among “Neurobiology of Disease,” “Molecular Neurobiology,” and “NPJ Parkinson’s Disease,” indicating the close connections of Parkinson’s disease transcriptomics research with fields such as neuroscience, molecular biology, genetics, bioinformatics, and clinical medicine. Analysis of co-cited journals shows that these journals have broad influence and knowledge dissemination within the research field.

A total of 1,181 authors have contributed to Parkinson’s disease transcriptomics research, with Glaab, Enrico being the most prolific author and Braak, H being the most highly cited. This facilitates the rapid identification of key figures and teams within the field, fostering interdisciplinary collaboration.

There are 12,183 co-cited references that have been widely cited, revealing the solid academic foundation of this research area. Additionally, a burst analysis of 10 documents reveals that they cover various aspects such as genetics, transcriptomics, cellular heterogeneity, disease progression, and data analysis, which are closely related to the molecular mechanisms, pathological processes, and potential therapeutic strategies of Parkinson’s disease. They also provide advanced data analysis tools, offering crucial technical support for Parkinson’s disease transcriptomics research. Keyword co-occurrence analysis shows that terms like “Parkinson’s Disease,” “Transcriptomics,” and “Neurodegeneration” have emerged over 15 times, highlighting their status as major research directions.

Cluster analysis further reveals five research directions in this field: (1) *Pathological mechanisms and disease progression*: Aging is a key risk factor for Parkinson’s disease. Metabolomics and neurotoxicity research explore the pathological basis from the perspectives of altered metabolic pathways and neuronal damage caused by neurotoxins. Network analysis reveals interactions between genes and proteins, jointly advancing the in-depth exploration of Parkinson’s disease pathology with neurodegenerative studies. (2) *Molecular—level research*: Proteomics and transcriptomics are key methods for elucidating gene expression and protein changes, while RNA sequencing focuses on alterations at the RNA level. These technologies collectively provide core support for unraveling the molecular mechanisms of Parkinson’s disease. Future research should integrate multi - omics data to comprehensively elucidate the molecular basis of the disease. (3) *Comparative studies and machine learning applications*: Alzheimer’s disease, Huntington’s disease, and Parkinson’s disease are common neurodegenerative diseases. Comparative studies help deepen the understanding of commonalities and differences between diseases. Machine learning applications aid in data analysis and the development of more accurate disease prediction and classification models. (4) *Neuroinflammation and specific proteins*: Alpha - synuclein is a key pathological protein in Parkinson’s disease. Microglia play an important role in neuroinflammation, which exacerbates disease progression. Transcriptomic studies, through high - throughput sequencing, analyze gene expression changes in brain tissues of Parkinson’s disease patients, helping to reveal disease mechanisms and identify biomarkers and therapeutic targets. (5) *RNA sequencing of specific brain regions*: RNA sequencing is a key technology for studying gene expression. The substantia nigra is one of the most severely affected regions in Parkinson’s disease. Related studies provide important region - specific information for understanding the disease. Future research should expand to other relevant brain regions and combine single - cell sequencing technologies to more precisely elucidate cell - specific changes in the disease.

These directions encompass a diverse exploration from molecular mechanism investigation to potential therapeutic strategy development. Moreover, trend analysis indicates that from 2014 to 2020, research focused on themes such as transcriptomics, Parkinson’s disease, and neurodegeneration, while after 2021, research began to expand into emerging fields such as specific proteins, spatial transcriptomics, single - cell RNA sequencing, and biomarker discovery. Notably, the research focus in Parkinson’s disease and transcriptomics has shifted from basic disease mechanisms to broader neurodegenerative diseases and multi - omics research areas.

### 4.2 Research status

Bibliometric methods provide a more objective and systematic approach to research analysis compared to traditional literature reviews. This methodology excels in its capacity to handle and analyze extensive datasets, discern patterns and trends, and offer a macro perspective on the research domain of Parkinson’s disease and transcriptomics. This comprehensive view allows for a more holistic understanding of the current research landscape. Consequently, leveraging prior literature, the authors have delineated the advancements in Parkinson’s disease and transcriptomics research across pivotal areas such as gene expression, biomarkers, pathogenic mechanisms, therapeutic strategies, drug responses and personalized medicine, disease model development, and the utilization of emerging technologies.

#### 4.2.1 Gene expression

Research emphasis is on critical brain regions including the substantia nigra, striatum, other forebrain areas, brainstem, and spinal cord, which are intimately connected with disease progression. Within the substantia nigra pars compacta, alterations in gene expression are associated with a pronounced reduction of dopaminergic neurons, indicative of neurodegenerative processes. The striatum, essential for dopaminergic signaling, shows gene expression changes linked to motor symptoms, whereas gene expression variations in forebrain regions like the frontal cortex correlate with cognitive impairments. Furthermore, gene expression shifts in the brainstem and spinal cord may be tied to the non-motor symptoms of Parkinson’s disease.

#### 4.2.2 Biomarkers

The field of biomarker research is burgeoning, with an ongoing emergence of novel findings and potential clinical applications. These investigations are augmenting our comprehension of the disease’s mechanisms and introducing innovative tools and methodologies for clinical practice. As technology progresses and research intensifies, it is projected that biomarkers will become increasingly pivotal in the diagnosis, management, and prevention of Parkinson’s disease. The current focus on biomarkers is summarized as follows:

The discovery of these biomarkers aids in a better understanding of the molecular mechanisms of Parkinson’s disease and may provide new tools for disease diagnosis, treatment, and prognosis assessment. However, many potential protein biomarkers still require further clinical research to validate their effectiveness and reliability. With the advancement of mass spectrometry technology, it is anticipated that more novel biomarkers will be discovered and verified (Table 7).

**Table 7:** Biomarkers and diagnostic tools in Parkinson’s disease research.

Biomarker	Description
α-synuclein	The abnormal aggregation of *α*-synuclein is one of the pathological hallmarks of Parkinson’s disease, and its mutations and overexpression are closely related to the onset and progression of the disease.
Dopamine Transporter	The reduction of DAT is associated with the loss of dopaminergic neurons and serves as a potential biomarker for the early diagnosis of Parkinson’s disease.
Cerebrospinal fluid	Alterations in the levels of certain proteins in CSF, such as a-synuclein, tau protein, and neurofilament light chain (NF-L), may act as biomarkers for Parkinson’s disease.
Gene mutation	Mutations in specific genes, such as LRRK2, PARK2, PARK7, etc., are associated with familial Parkinson’s disease.
Mitochondrial dysfunction	Mutations in mitochondrial DNA and mitochondrial dysfunction are related to the pathogenesis of Parkinson’s disease.
Oxidative stress	Markers such as 8-OHdG and MDA indicate oxidative stress, which plays a role in the disease’s progression.
Neuroinflammation	Neuroinflammation is associated with the advancement of Parkinson’s disease, and certain inflammatory factors in CSF may serve as indicators of disease activity.
Metabolomics	Metabolomic studies have revealed changes in metabolites associated with Parkinson’s disease, such as alterations in amino acids, lipids, and energy metabolism.
Proteomics	Proteomic analyses have identified changes in protein expression associated with Parkinson’s disease, including synaptic proteins like Synapsin I and Synaptophysin, and alterations in autophagy-related proteins.
Imaging techniques	PET imaging of the dopaminergic system (e.g., DAT-SPECT) and MRI technology can serve as auxiliary diagnostic tools for Parkinson’s disease.

#### 4.2.3 Pathological mechanisms

Current research focuses on mechanisms such as the degenerative changes in dopaminergic neurons, *α*-synuclein aggregation, mitochondrial dysfunction, oxidative stress, neuroinflammation, protein homeostasis imbalance, neuro transmission and synaptic dysfunction, cellular autophagy and cell death, and perturbations in intercellular communication and network disturbances. As transcriptomics technology continues to advance, future research will more precisely elucidate the changes in these pathological mechanisms.

#### 4.2.4 Therapeutic strategies

Research on therapeutic strategies is progressing with the aim of improving treatment efficacy for Parkinson’s disease, delaying disease progression, and enhancing the quality of life for patients. Treatment methods include: (1) Dopamine replacement therapy: Enhancing dopamine levels by supplementing dopamine precursors or agonists to improve motor symptoms. (2) Neuroprotective strategies: Protecting dopaminergic neurons from degenerative effects using brain-derived neurotrophic factor (BDNF) and glial cell line-derived neurotrophic factor (GDNF). (3) Anti-inflammatory treatment: Exploring anti-inflammatory drugs or small molecules to reduce neuroinflammation and protect neurons. (4) Cellular therapy: Utilizing stem cell or neural precursor cell transplantation to replace lost neurons. (5) Gene therapy: Applying gene-editing techniques (such as CRISPR/Cas9) or RNA interference technology to modulate gene expression related to the disease. (6) Immunotherapy: Exploring the use of immunomodulators or immune checkpoint inhibitors to influence the immunopathological processes of PD. (7) Cellular autophagy and protein clearance mechanisms: Clearing abnormal protein aggregates. (8) Mitochondrial protective agents: Improving mitochondrial function and reducing oxidative stress.

#### 4.2.5 Drug response and personalized medicine

Due to significant individual differences in clinical manifestations, disease progression, and response to drug treatment among Parkinson’s disease patients, research on drug response and personalized medicine is of paramount importance. Pharmacogenomic studies can identify genetic markers and polymorphisms associated with responses to anti-Parkinson’s disease drugs to predict individual responses. Personalized medicine is primarily reflected in providing customized treatment plans based on patients’ genotypic and phenotypic differences. Additionally, the application of single-cell transcriptome sequencing technology helps understand the heterogeneity of different cellular subsets in Parkinson’s disease, offering new perspectives for disease classification, diagnosis, and treatment.

#### 4.2.6 Disease model establishment

Existing literature indicates that human-induced pluripotent stem cells (iPSCs) technology can be used to differentiate into disease-relevant cell types for the establishment of *in vitro* disease models and drug screening. The popular single-cell RNA sequencing technology (scRNA-seq) reveals cellular heterogeneity among dopaminergic neurons in Parkinson’s disease, aiding in understanding disease mechanisms and therapeutic strategies. Animal models can also be induced using neurotoxins such as 6-OHDA and MPTP to mimic the pathological processes of Parkinson’s disease. Furthermore, gene-editing techniques (such as CRISPR/Cas9) can introduce Parkinson’s disease-related gene mutations into cells to establish disease models, helping to study the impact of specific genetic variations on disease phenotypes.

#### 4.2.7 Application of emerging technologies

With the advancement of economic standards and technology, the development of emerging technologies provides new perspectives and tools for understanding disease mechanisms, diagnosis, and treatment. The authors have reviewed some key emerging technologies involved in the field of Parkinson’s disease and transcriptomics research, hoping to bring breakthrough progress in the treatment of Parkinson’s disease (Table 8).

**Table 8:** Emerging technologies in Parkinson’s disease research and their applications.

Emerging technology	Description
Single-cell RNA Sequencing	Enables gene expression analysis at the single-cell level, revealing cellular heterogeneity and state transitions, enhancing understanding of cellular specificity changes in Parkinson’s disease.
Spatial Transcriptomics	Combines spatial information of tissues with gene expression analysis, aiding in understanding the role of cells within their microenvironment and improving knowledge of cellular spatial distribution during Parkinson’s disease pathology.
Long-read Sequencing Technology	Provides more complete genomic structure information, facilitating the identification of complex genetic variations, and contributing to the understanding of the genomic structure in Parkinson’s disease.
CRISPR/Cas9 Gene Editing Technology	Used for precise gene modification to study the role of specific genes in Parkinson’s disease, enhancing the precision of gene function research.
Artificial Intelligence and Machine Learning	Analyzes large-scale transcriptomic data, identifies biomarkers and therapeutic targets, and optimizes the efficiency and accuracy of data analysis.
Multi-omics Integration Analysis	Combines various omics data to provide a comprehensive disease profile, aiding in the understanding of the complex biological characteristics of Parkinson’s disease.
Microfluidics and Single-cell Analysis Technology	Conducts high-throughput experiments at the single-cell level, enhancing in-depth understanding of cellular functions and interactions.
Optogenetics	Uses light-controlled ion channels to modulate neuronal activity, studying changes in neural circuits, and researching the neuroregulatory mechanisms of Parkinson’s disease.
Nanotechnology	Has potential applications in drug delivery and biomarker detection, improving the precision and efficiency of treatment.
Wearable Devices and Mobile Health Technology	Monitors patients’ physiological and motor parameters in real-time, providing data support for disease management.
3D Cell Culture and Organ-on-a-chip Technology	Simulates the human microenvironment for the establishment of disease models and drug screening.
Computational and Systems Biology Approaches	Utilizes computational models to simulate disease progression and mechanisms of drug action, predicting disease development and treatment responses.

### 4.3 Critical evaluation of research gaps in Parkinson’s disease transcriptomics

While transcriptomic studies have provided valuable insights into the molecular mechanisms of PD, several critical research gaps remain unaddressed. One of the most significant challenges is the lengthy and high-failure-rate process of biomarker validation. Despite numerous promising biomarkers identified in laboratory settings, their translation to clinical applications is often hindered by the lack of robust validation frameworks and standardized methodologies. Biomarker validation requires multi-step processes, including preclinical testing, clinical trials, and regulatory approval, each of which presents unique challenges and high attrition rates (Hussein et al., 2023; Kim et al., 2014).

Another critical issue is the heterogeneity of transcriptomic data across studies. Differences in sample collection, RNA extraction protocols, and data analysis methods lead to inconsistent results, making it difficult to compare findings across studies and hindering the validation of potential biomarkers in independent cohorts (Huang et al., 2021; Alharbi et al., 2024). This heterogeneity is further exacerbated by small sample sizes, which fail to capture the full spectrum of disease variability and limit the statistical power of analyses.

Moreover, the lack of standardized experimental protocols and analytical frameworks remains a significant barrier to progress. While methods for integrating single-cell RNA sequencing with spatial transcriptomics have advanced, fewer tools exist for incorporating epigenomic data, which could provide critical insights into regulatory mechanisms underlying disease pathogenesis (Luo et al., 2024; Danishuddin and Kim, 2024). The development of novel computational tools and standardized workflows is essential to address these challenges and enhance the comparability and reproducibility of transcriptomic studies.

A notable gap lies in the underrepresentation of non-motor symptom (NMS) research in PD transcriptomics. Over 80% of studies focus on motor symptom pathways, despite the profound impact of NMS—such as cognitive impairment, mood disorders, sleep disturbances, and autonomic dysfunction—on patient quality of life. For example, plasma orexin-A concentrations have been linked to NMS severity in PD patients, suggesting its potential as a biomarker, yet the molecular pathways driving these associations remain unexplored (Huang et al., 2021). Similarly, dysregulation of GABAergic neurotransmission has been implicated in both motor and non-motor symptoms, but transcriptomic studies rarely investigate these pathways in the context of NMS (Alharbi et al., 2024). Genetic factors, such as LRRK2 variants, further complicate NMS progression, yet transcriptomic analyses of genotype–phenotype interactions in non-motor domains are scarce (Deng et al., 2019). Additionally, the lateralization of motor symptoms has been shown to influence non-motor outcomes post-intervention (e.g., deep brain stimulation), highlighting the need for integrated studies examining spatial transcriptomic patterns and their relationship to symptom asymmetry (Voruz et al., 2022).

Future research should focus on overcoming these limitations by prioritizing the development of robust biomarker validation frameworks, improving standardization of experimental protocols, and advancing computational tools for multi-omics data integration. Expanding research to address underrepresented areas is critical, for instance, integrating epigenomic data to explore regulatory mechanisms of NMS, leveraging spatial transcriptomics to map cellular interactions in brain regions associated with cognitive or autonomic dysfunction, and conducting large-scale longitudinal studies to capture dynamic transcriptomic changes linked to NMS progression. Such efforts will provide a more comprehensive understanding of PD’s molecular landscape and pave the way for personalized therapeutic strategies that address both motor and non-motor dimensions of the disease.

### 4.4 Future research directions

**Figure 7:**
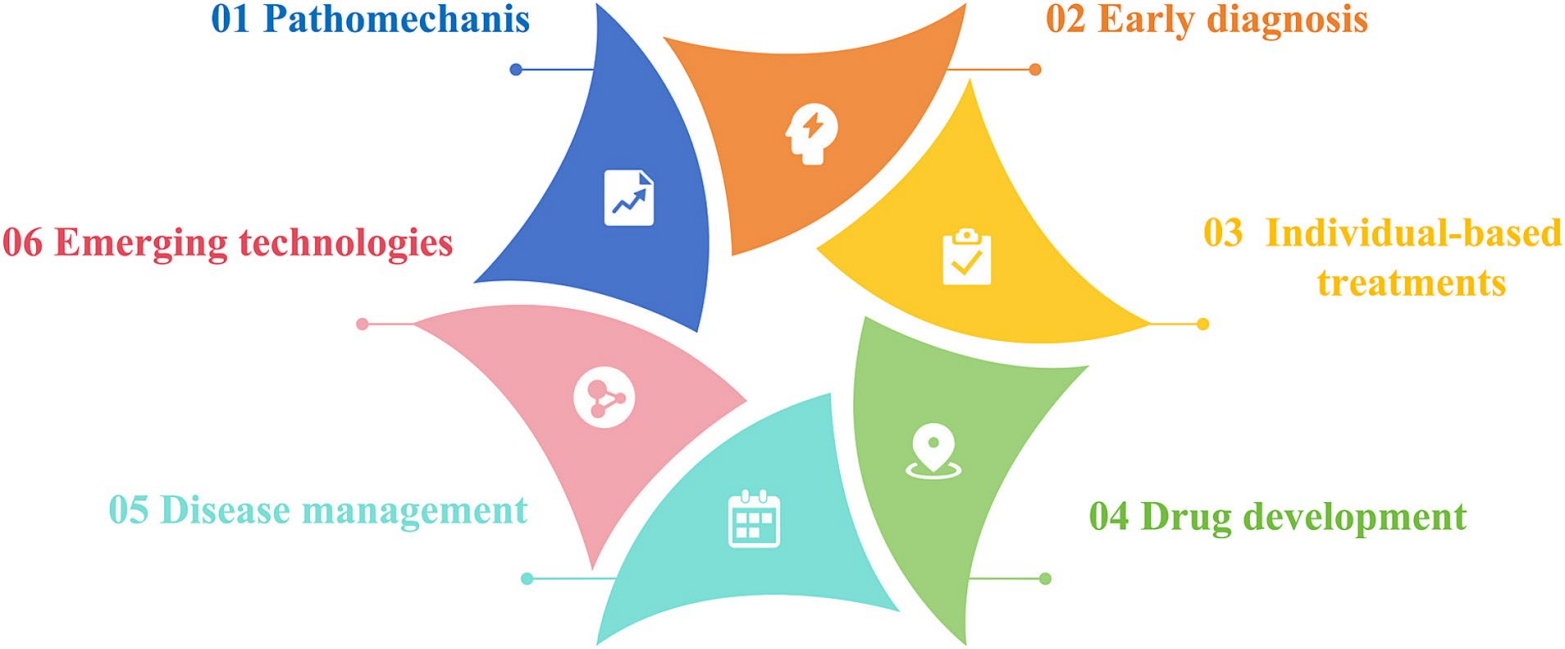
A synthesized overview of the research directions in the field of Parkinson’s disease transcriptomics.

The field of Parkinson’s disease and transcriptomics is rapidly advancing, encompassing the entire spectrum from fundamental molecular mechanism exploration to clinical treatment applications. This underscores the importance of interdisciplinary collaboration and the potential of emerging technologies to propel research and therapeutic advancements. As these technologies progress and are applied, they are expected to bring groundbreaking progress to various research directions in the field of Parkinson’s disease and transcriptomics, including pathological mechanisms, early diagnosis, personalized treatment, drug development, disease management, and emerging technologies. The specifics are as follows (Figure 7).

#### 4.4.1 Pathological mechanisms

Multi-omics integration: Combine spatial transcriptomics with epigenomic, proteomic, and metabolomic datasets to unravel cell-type-specific pathways (e.g., dopaminergic neuron vulnerability) and microenvironmental interactions (e.g., neuroinflammation in the substantia nigra) (Titova et al., 2017; Dorsey et al., 2018; Luo et al., 2024; Danishuddin and Kim, 2024).

NMS focus: Expand beyond motor-centric studies to map transcriptomic changes in brain regions linked to cognitive impairment (e.g., prefrontal cortex) and autonomic dysfunction (e.g., locus coeruleus), leveraging single-nucleus RNA-seq to resolve cellular subpopulations (Bloem et al., 2021; Armstrong and Okun, 2020; Keo et al., 2021; Fereshtehnejad et al., 2019).

#### 4.4.2 Early diagnosis

Liquid biopsy development: Validate minimally invasive biomarkers (e.g., plasma orexin-A, exosomal miRNAs) through longitudinal cohort studies, integrating AI-driven analysis of transcriptomic data with wearable device metrics (Bloem et al., 2021; Huang et al., 2021; Voruz et al., 2022).

Pre-symptomatic detection: Investigate transcriptomic signatures in prodromal PD cohorts (e.g., REM sleep behavior disorder patients) to identify predictive biomarkers (Fereshtehnejad et al., 2019; Deng et al., 2019).

#### 4.4.3 Personalized treatment

Pharmacogenomic stratification: Use transcriptomic profiles to predict drug responses (e.g., L-DOPA efficacy fluctuations) and tailor therapies for genetic subtypes (e.g., *LRRK2* or *GBA* mutation carriers)(Keo et al., 2021; Deng et al., 2019; Lang et al., 2019).

NMS-targeted therapies: Develop interventions addressing GABAergic dysregulation or orexinergic signaling imbalances identified through transcriptomic studies of non-motor pathways (Bloem et al., 2021; Armstrong and Okun, 2020; Alharbi et al., 2024).

#### 4.4.4 Drug development

High-throughput screening: Apply CRISPR-based transcriptomic perturbation screens to identify novel targets (e.g., neuroprotective genes in glial cells) and repurpose existing drugs (Lang et al., 2019; Kriks et al., 2011).

Organoid and animal models: Validate targets using patient-derived iPSC models with spatial transcriptomic resolution to mimic PD pathology *in vitro* (Agarwal et al., 2020; Smajić et al., 2022).

#### 4.4.5 Disease management

Digital health integration: Build federated databases linking transcriptomic data with real-world outcomes (e.g., DBS efficacy, cognitive decline trajectories) to enable AI-powered predictive models (Fereshtehnejad et al., 2019; Voruz et al., 2022).

Global patient registries: Incorporate diverse ethnic and socioeconomic cohorts to ensure biomarker and therapeutic applicability across populations (Nalls et al., 2019; Blauwendraat et al., 2020).

#### 4.4.6 Emerging technologies

Spatial multi-omics: Deploy MERFISH or Slide-seq to map RNA-protein interactions in PD-affected tissues, uncovering spatially resolved regulatory networks (Titova et al., 2017; Dorsey et al., 2018; Bellou et al., 2016).

AI-driven drug discovery: Train deep learning models on transcriptomic datasets to predict drug-disease interactions and optimize combinatorial therapies (Armstrong and Okun, 2020; Ma et al., 2021; Danishuddin and Kim, 2024).

Ethical and accessible innovation: Ensure equitable access to advanced technologies (e.g., low-cost single-cell sequencing platforms) in resource-limited settings (Mattsson-Carlgren et al., 2020; Priem et al., 2010).

## 5 Limitations of research

Our bibliometric study has the following limitations that warrant cautious interpretation of the results:

### 5.1 Language and database coverage bias

The exclusive reliance on English-language literature from the Web of Science Core Collection may introduce selection bias. Publications in non-English journals (e.g., Chinese, German) or regional databases (e.g., CNKI, SciELO) were excluded, potentially underrepresenting contributions from non-Anglophone countries. Additionally, the WoSCC’s journal inclusion criteria favor established Western journals, which may overlook emerging research from developing regions.

### 5.2 Temporal and citation biases

Recently published high-impact studies (e.g., spatial transcriptomics articles post-2022) are likely underrepresented due to the “citation lag” phenomenon, as novel findings require time to accumulate citations. Conversely, older publications with methodological limitations might be overrepresented due to prolonged citation cycles.

### 5.3 Metric-driven distortions

The reliance on citation-based metrics (e.g., citation counts, H-index) inherently prioritizes studies from high-profile journals or influential teams, amplifying the “Matthew effect” in academic visibility. While these metrics are widely used, they may inadequately reflect scientific quality or clinical relevance. For instance, methodologically rigorous studies—including those reporting negative results (e.g., failed biomarker validations (Mattsson-Carlgren et al., 2020)) or incremental technical advances—often receive limited citations compared to hypothesis-driven “hot topics” (e.g., *α*-synuclein). This bias is further exacerbated by preferential citation practices within high-impact journal networks and self-citation cartels, which artificially inflate the perceived significance of certain works. To address these limitations, future bibliometric studies should triangulate traditional citation data with alternative metrics such as altmetrics (Priem et al., 2010) (e.g., social media engagement), clinical trial registrations, and mentions in clinical guidelines. Such an integrative approach would provide a more balanced assessment of translational impact and real-world relevance, ensuring that both foundational methodological contributions and clinically actionable findings are appropriately recognized.

### 5.4 Technical heterogeneity

Transcriptomic methodologies (e.g., bulk RNA-seq vs. single-cell sequencing) vary significantly across studies, complicating direct comparisons. Platform-specific biases (e.g., 3′-end bias in scRNA-seq) and batch effects were not accounted for in our analysis, potentially masking critical technical confounders.

## 6 Conclusion

This study represents the first comprehensive bibliometric analysis of transcriptomics research in PD, systematically mapping the evolution of knowledge structures, research hotspots, and global contributions in this field. By analyzing 208 publications spanning from 2011 to 2025, we identified a surge in research activity since 2020, driven by advancements in single-cell sequencing, spatial transcriptomics, and multi-omics integration. Key journals such as Nature Communications and NPJ Parkinson’s disease emerged as central hubs for knowledge dissemination, while institutions in the United States and China demonstrated leading productivity. Keyword and citation analyses further highlighted growing interests in neuroinflammation, biomarker discovery, and machine learning applications, reflecting a shift from mechanistic studies toward translational and interdisciplinary approaches.

Despite these advancements, critical challenges remain. Our analysis underscores persistent heterogeneity in transcriptomic data across studies, stemming from inconsistent experimental protocols and analytical frameworks. Such variability complicates biomarker validation—a process already hindered by high attrition rates in clinical translation. Additionally, reliance on citation-based metrics may inadvertently amplify the visibility of hypothesis-driven studies over methodologically rigorous but less-cited work, such as negative results or technical validations. These limitations highlight the need for standardized protocols, robust validation pipelines, and alternative impact metrics that prioritize clinical relevance.

Looking forward, we propose three strategic directions to propel the field:

### 6.1 Technological integration

Combining single-cell and spatial transcriptomics with epigenomic and proteomic datasets will provide a holistic view of PD pathology, enabling the identification of cell-type-specific pathways and microenvironmental interactions. For instance, leveraging spatial transcriptomics to map cellular dynamics in brain regions linked to non-motor symptoms (e.g., locus coeruleus for autonomic dysfunction) could uncover novel regulatory networks.

### 6.2 Global Collaboration

Establishing multinational consortia to harmonize data collection, share biospecimens, and validate biomarkers across diverse cohorts is essential to mitigate data fragmentation and enhance reproducibility. Emphasis should be placed on underrepresented populations (e.g., Asian or African cohorts) to address genetic and phenotypic heterogeneity.

### 6.3 Clinical translation

Prioritizing studies that link transcriptomic signatures to longitudinal clinical outcomes—such as non-motor symptom progression (e.g., cognitive decline, sleep disturbances) or deep brain stimulation efficacy—will bridge the gap between bench discoveries and bedside applications. Emerging tools like wearable devices and AI-driven analytics offer unprecedented opportunities to contextualize omics data within real-world patient trajectories.

By addressing these challenges through interdisciplinary innovation and international cooperation, PD transcriptomics can transition from a rapidly expanding field to one that delivers actionable insights for early diagnosis, personalized therapies, and ultimately, disease modification. The integration of cutting-edge technologies with clinically grounded frameworks will be pivotal in transforming scientific promise into tangible patient benefits.
